# Equine poisoning by coffee husk (*Coffea arabica *L.)

**DOI:** 10.1186/1746-6148-8-4

**Published:** 2012-01-12

**Authors:** Diego Jose Z Delfiol, Jose P Oliveira-Filho, Fernanda L Casalecchi, Thatiane Kievitsbosch, Carlos A Hussni, Franklin Riet-Correa, João P Araujo-Jr, Alexandre S Borges

**Affiliations:** 1 Department of Veterinary Clinical Science, College of Veterinary Medicine and Animal Science - Univ Estadual Paulista (UNESP), Botucatu, São Paulo, 18618970, Brazil; 2 Department of Veterinary Clinical Science, College of Veterinary Medicine and Animal Science - Univ Estadual Paulista (UNESP), Botucatu, São Paulo, 18618970, Brazil; 3 Faculdade de Medicina Veterinária - Fundação Pinhalense de Ensino, Espírito Santo do Pinhal, São Paulo, 13990000, Brazil; 4 Department of Veterinary Clinical Science, College of Veterinary Medicine and Animal Science - Univ Estadual Paulista (UNESP), Botucatu, São Paulo, 18618970, Brazil; 5 Department of Veterinary Surgery and Anesthesiology, College of Veterinary Medicine and Animal Science - Univ Estadual Paulista (UNESP), Botucatu, São Paulo, 18618970, Brazil; 6 Hospital Veterinário - Campus de Patos da Universidade Federal de Campina Grande, Patos, Paraíba. 58700000, Brasil; 7 Department of Immunology and Microbiology, Biosciences Institute of Botucatu - Univ Estadual Paulista (UNESP), Botucatu, São Paulo, 18618000, Brazil; 8 Department of Veterinary Clinical Science, College of Veterinary Medicine and Animal Science - Univ Estadual Paulista (UNESP), Botucatu, São Paulo, 18618970, Brazil

## Abstract

**Background:**

In Brazil, coffee (*Coffea arabica*) husks are reused in several ways due to their abundance, including as stall bedding. However, field veterinarians have reported that horses become intoxicated after ingesting the coffee husks that are used as bedding. The objective of this study was to evaluate whether coffee husk consumption causes intoxication in horses.

**Results:**

Six horses fed coast cross hay *ad libitum *were given access to coffee husks and excitability, restlessness, involuntary muscle tremors, chewing movements and constant tremors of the lips and tongue, excessive sweating and increased respiration and heart rates were the most evident clinical signs. Caffeine levels were measured in the plasma and urine of these horses on two occasions: immediately before the coffee husks were made available to the animals (T0) and at the time of the clinical presentation of intoxication, 56 h after the animals started to consume the husks (T56). The concentrations of caffeine in the plasma (p < 0.001) and urine (p < 0.001) of these animals were significantly greater at T56 than at T0.

**Conclusions:**

It was concluded that consumption of coffee husks was toxic to horses due to the high levels of caffeine present in their composition. Therefore, coffee husks pose a risk when used as bedding or as feed for horses.

## Background

Brazil is the largest coffee producer in the world, and coffee grain processing generates a large amount of waste, given that close to 60% of the crude weight of the bean corresponds to the husk. The coffee (*Coffea arabica*) husk is rich in organic compounds and contains substances such as tannins, polyphenols and caffeine; the latter is often found in higher concentrations in the husk than in the bean [1]. Several studies have addressed reuse of the husks, especially as organic fertilizers, in tea production, caffeine extraction and the feeding of ruminants and pigs [2-6].

Caffeine (C_8_H_10_N_4_O_2_) is a methylxanthine, which the most important effect is adenosine receptor antagonism [7]. Adenosine reduces spontaneous neuronal firing in multiple brain areas, and its presynaptic action inhibits neuronal release of acetylcholine, norepinephrine, dopamine, serotonin and gamma-aminobutyric acid; it produces sedation and has an anticonvulsant effect [8]. When caffeine binds to adenosine receptors, it prevents the inhibitory effects of adenosine [8], thereby exerting an excitatory action on smooth muscles and on several body systems, including the central nervous system, cardiorespiratory system and gastrointestinal system [9-11]. Another described caffeine effect is the inhibition of phosphodiesterases, capable of cAMP inactivation. This resultant cAMP accumulation stimulates catecholamines actions [12,13].

Found mainly in coffee, teas, soft drinks, energy drinks, chocolate and in some medications [14,15], caffeine is considered a leading cause of intoxication in humans, and several fatal cases have been reported [16-19]. Natural cases of intoxication have also been described in cats and dogs [15]; furthermore, lethal doses of caffeine combined with theobromine are used for population control in coyotes [20].

Experimental intoxication by coffee husks has been described in cattle in Brazil, and the signs of hyperexcitability, muscle tremors, increased heart and respiratory rates, spontaneous falls, opisthotonus and seizures were attributed to the caffeine found in the husks [21].

In horses, there are no reports of intoxication by coffee husks described in the literature. Nevertheless, at a stud farm (22° 11' 27" S, 46° 44' 27'' W) located in São Paulo, Brazil, one of the authors (Casalecchi F.L.) treated a horse that was stabled for two days in a stall with coffee husk bedding and that showed signs of hyperexcitability, excessive sweating, involuntary movements of the lips and tongue, increased heart and respiratory rates and difficulty in grasping forage; these clinical signs ceased 24 h after access to coffee husks was restricted. Professionals working in the region report that similar problems have been observed at stud farms that use coffee husks for bedding; the problems have been solved with removal of the bedding. These reports suggest that coffee husks are toxic to horses. Therefore, the objective of this study was to evaluate whether spontaneous coffee husk consumption by horses is able to cause clinical signs of intoxication.

## Methods

### Experimental animals

To be included in this research, the horses underwent physical and laboratory tests (complete blood count, plasma fibrinogen level, and venous blood gas). Animals presenting no changes at the clinical examination and with laboratory test values within the normal range for the species were considered to be healthy, and six Quarter Horse mares, not pregnant, with an average age of 10 ± 2 years and average weight of 425 ± 32 kg, were selected to participate in the study. The animals were dewormed and underwent a 15-day adaptation period before coffee husks were offered.

These animals were kept in individual 12-m^2 ^stalls and were fed coast cross (*Cynodon dactylon*) hay and water *ad libitum*. All animals were their own controls. The procedures performed were previously approved by the Ethics Committee on Animal Use of the School of Veterinary Medicine and Animal Science, Univ. Estadual Paulista (CEUA-89/2010).

### Analysis of coffee husks

The coffee husks used in the experiment were obtained from a single milling process, from the Coffee Growers Cooperative of São Manoel, in the state of São Paulo. After the coffee husks were homogenized, samples were submitted for analysis to a commercial laboratory prior to being administered to the horses. These analyses consisted of: (i) determination of caffeine, theobromine and theophylline content by high-performance liquid chromatography (HPLC); (ii) qualitative determination of insecticides by silica gel thin layer chromatography (chloride, carbamate and phosphate compounds); and (iii) determination of mycotoxin concentrations (fumonisins B1 and B2, ochratoxin A and aflatoxins B1, B2, G1 and G2) by HPLC.

### Administration of coffee husks

Animals were supplied with coffee husks right after the adaptation period, as follows. On day 1, an additional trough containing 2 kg of coffee husks was introduced into each stall. Every 12 h, the surplus was removed and weighed, the value was recorded in individual records of consumption, and another 2 kg of husks was subsequently placed in the trough. Hay and water were supplied *ad libitum *throughout the experimental period. The animals were continuously observed and subjected to clinical examination immediately before the administration of coffee husks and then every 12 h through 120 h (T120), as well as when neurological abnormalities were exhibited (T56) (with subsequent discontinuation of coffee husk administration from that point on).

### Caffeine concentration in biological samples

Plasma and urine caffeine concentrations were measured at T0 and T56, using plasma and urine samples. Blood samples were collected in tubes containing sodium heparin by venipuncture of the jugular vein with a 21-G needle, and the plasma was separated by centrifugation, while urine samples were collected using a urinary catheter. Caffeine concentrations were measured in duplicate by competitive enzyme-linked immunosorbent assay (ELISA) (Racing ELISA kit for Caffeine/Pentoxifylline - Neogen^®^, Lexington, KY, USA), following the manufacturer's recommendations. The plates were subjected to light absorption readings at 450 nm optical density (OD) in an automated ELISA reader (Multiskan EX - Labsystems^®^, Bucharest, Romania). To determine the quantitative plasma and urine concentrations of caffeine, a curve was plotted using a known caffeine concentration standard (Caffeine solution C6035 - Fluka^® ^Analytical, St. Louis, MO, USA), which varied from 1 to 100 ng/ml; the OD value found in the ELISA test for each sample was subjected to a regression analysis to determine the caffeine concentration in ng/ml. Plasma and urine samples with a caffeine concentration of more than 100 ng/ml were serially diluted in phosphate-buffered saline until the concentration fit the standard curve.

### Clinical chemistry

Commercial colorimetric kits were used to determine the serum concentrations of total protein (TP) (Biuret Test - Katal^®^, Belo Horizonte, Brazil), albumin (Bromocresol Green - Katal^®^), urea (Urease Test, Berthelot - Katal^®^), creatinine (Jaffé Test - Katal^®^) and iron (Chromazurol - Laborlab^®^, Guarulhos, Brazil) and the serum activities of creatine kinase (CK) (Kinetic UV Test - Katal^®^), aspartate aminotransferase (AST) (Kinetic UV Test - Katal^®^) and gamma-glutamyltransferase (GGT) (Gamma-GT/Kinetic Test - Katal^®^). The tests were performed following the manufacturer's recommendations, and the results were analyzed using an automated biochemical analyzer (SB190 - Celm^®^, Barueri, Brazil). The serum globulin concentrations were calculated by the difference between total protein and albumin. For these measurements, blood samples were collected in tubes without anti-coagulants, at the same time periods described previously. After observing clot retraction, the samples were immediately centrifuged, and the resulting serum was stored in plastic tubes and frozen at -20°C until the biochemical tests were processed.

### Blood gas parameters

The venous blood gas analysis (blood pH, hematocrit [Htc], hemoglobin [Hb], partial pressure of CO_2 _[PCO_2_], partial pressure of O_2 _[PO_2_], total CO_2 _concentration [tCO_2_], oxygen saturation [SO_2_], base excess and concentrations of HCO_3_, Na, K and ionized calcium [iCa]) (i-STAT EG7^+® ^- Abbott^®^, East Windsor, NJ, USA) was conducted before providing and immediately after discontinuing the coffee husk supply. For this reason, 1 ml of venous blood was withdrawn with insulin syringes containing 200 IU of sodium heparin. The blood gas parameters were measured immediately after blood collection, using a portable blood analyzer (i-STAT^® ^1 Analyzer - Abbott^®^). The serum concentration of the chloride ion was measured using a colorimetric kit (Quimicloro/colorimetric method - EBRAM, São Paulo, Brazil), following the manufacturer's recommendations, and the results were analyzed using an automated biochemical analyzer (SB-190 - Celm^®^).

### Statistical analysis

The results obtained at each time period generated arithmetic means and standard deviations, and they were subjected to statistical analysis using the GraphPad InStat for Windows program, version 3.0 (GraphPad Software). Heart rate, respiratory rate and rectal temperature results were assessed by analysis of variance (ANOVA), with Tukey's post-hoc test. The results of the biochemical tests and blood gas analyses and caffeine concentration were compared between T0 and T56 by a paired t-test. Statistical differences were considered to be significant at p ≤ 0.05.

## Results

Analysis of the coffee husk samples did not reveal the presence of insecticides or mycotoxins, and the concentration of caffeine found was 0.9%. Theobromine and theophylline were not detected. Coffee husk consumption by the animals was spontaneous throughout the experimental period, and the husks were removed when the animals displayed clinical signs of intoxication (T56), at which time a clinical examination was also performed on the animals.

A small amount of coffee husks was consumed in the first 12 h, and then consumption increased (Table 1). After 48 h, all animals were more hyperresponsive to sound stimuli produced in the environment. Tachycardia (106 ± 52 beats per minute - bpm) and tachypnea (27 ± 8 movements per minute - mpm) peaks were detected at T56 and were significantly higher than T0 levels, p < 0.001 and p < 0.01 respectively. The mean cardiac rate was also significantly higher than T0 levels (35 ± 5 bpm) at T60 (85 ± 41 bpm, p < 0.05) and T72 (79 ± 43 bpm, p < 0.05), while mean respiratory rate was significantly higher than T0 levels (12 ± 4 mpm) between T48 (27 ± 11 mpm, p < 0.01) and T60 (25 ± 4 mpm, p < 0.05). No cardiac rhythm abnormalities were observed during auscultation.

**Table 1:** Amount of coffee husks (g) and caffeine* (g) consumed per animal over the evaluated times

	Experimental time (hours)											
	T12		T24		T36		T48		T56		Total	
	
Animal	***husks***	***caffeine***	***husks***	***caffeine***	***husks***	***caffeine***	***husks***	***caffeine***	***husks***	***caffeine***	***husks***	***caffeine***
**1**	70	0.6	1,300	11.7	1,800	16.2	1,300	11.7	40	0.4	4,510	40.6
**2**	70	0.6	1,640	14.8	2,000	18.0	1,100	9.9	270	2.4	5,080	45.7
**3**	20	0.2	1,800	16.2	1,900	17.1	1,600	14.4	60	0.5	5,380	48.4
**4**	110	1.0	700	6.3	1,000	9.0	900	8.1	15	0.1	2,725	24.5
**5**	40	0.4	560	5.0	1,350	12.2	350	3.2	0	0.0	2,300	20.7
**6**	30	0.3	470	4.2	650	5.9	700	6.3	20	0.2	1,870	16.8

**Mean**	57	0.5	1,078	9.7	1,450	13.1	992	8.9	68	0.6	3,644	32.8
**St. Dev**.	33	0.3	578	5.2	544	4.9	443	4.0	101	0.9	1,524	13.7

*The concentration of the caffeine consumed was calculated according to the amount of coffee husks consumed X the caffeine concentration in the husks (0.9%).

The mean rectal temperature was statistically higher compared with the T0 (37.2 ± 0.2°C) between T56 (38.9 ± 0.7°C, p < 0.001) and T84 (38.3 ± 0.3°C, p < 0.01). Furthermore, four animals (1, 2, 3 and 5) were restless, with muscle tremors, mydriasis, congested ocular mucosa and episcleral vessels, excessive sweating and decreased intestinal motility. The involuntary tremor of the lips and constant movement of the lips and tongue were severe in animals 1 and 5 (Additional File 1) and were mild in animals 2 and 3. Animals 4 and 6 displayed mild tachycardia and did not show signs of muscle tremors or sweating. At T56, the supply of coffee husks was discontinued, and the clinical signs gradually decreased and then ceased after 12 h in animals 3, 4 and 6 and in after 40 h in animals 1, 2 and 5; at the end of the experiment (T120), the animals did not show any noticeable clinical abnormalities.

The amount of caffeine ingested by each animal up to the onset of clinical signs was estimated using data on coffee consumption and the caffeine concentration in the husk (Table 1). The results of the biochemical analyses are provided in Table 2. The mean values for TP, albumin, globulin, AST and creatinine were significantly different between T0 and T56. The blood gas values of PCO_2_, HCO_3_, tCO_2_, SO_2_, K and iCa were significantly different between T0 and T56, relative polycythemia was observed in all animals, and Htc and Hb increased by 47% and 50%, respectively, at T56 relative to T0 (Table 3). There was no statistical difference between the average values of serum chloride in the animals at T0 (109 ± 2 mmol/l) and T56 (109 ± 3 mmol/l). Table 4 shows the plasma and urine caffeine values obtained for the horses at T0 and T56.

**Table 2:** Mean and standard deviation of serum concentrations variables of horses (n = 6) subjected to intoxication by coffee husk, at T0 and T56

Times	*TP* *(g/dl)*	*Albumin* *(g/dl)*	*Globulin* *(g/dl)*	*GGT* *(IU/l)*	*AST* *(IU/l)*	*CK* *(IU/l)*	*Urea* *(g/dl)*	*Creatinine* *(g/dl)*	*Iron* *(μg/dl)*
**T0**	6.2 ± 0.4	3.1 ± 0.1	3.1 ± 0.4	61.7 ± 7.5	148 ± 48	253 ± 34	39 ± 6	1.3 ± 0.3	111 ± 24
**T56**	6.9 ± 0.9	3.2 ± 0.1	3.7 ± 0.8	73.3 ± 16.3	274 ± 44	650 ± 439	34 ± 4	2.1 ± 0.4	98 ± 27
p*	0.0176	0.0182	0.0313	ns	0.0313	ns	ns	0.0004	Ns

* A value of p ≤ 0.05 represented a statistically significant difference between T0 and T56.

**Table 3:** Mean and standard deviation of blood gas, hematocrit and hemoglobin variables in six horses subjected to intoxication by coffee husks at T0 and T56

Times	pH	PCO_2 _(mm Hg)	PO_2 _(mm Hg)	BE (mmol/l)	HCO_3 _(mmol/l)	tCO_2 _(mm Hg)	SO_2 _(%)	Na (mmol/l)	K (mmol/l)	iCa (mmol/)	Htc (%)	Hb (g/dl)
**T0**	7.4 ± 0	46.7 ± 3.25	29.5 ± 3.1	3.8 ± 2.3	28.5 ± 2.34	30 ± 2.28	52.8 ± 7.7	137.0 ± 1.1	4.0 ± 0.14	1.7 ± 0.04	30 ± 3	10 ± 1.1
**T56**	7.37 ± 0.07	36.4 ± 4.17	41.2 ± 13.5	-3.4 ± 6.7	21.4 ± 5.71	22.4 ± 5.85	67.2 ± 11.5	138.7 ± 2.34	3.4 ± 0.56	1.5 ± 0.09	44 ± 5	15 ± 1.6
p*	ns	0.01	ns	ns	0.04	0.04	0.01	ns	0.04	0.002	0.004	0.004

* A value of p ≤ 0.05 represented a statistically significant difference between T0 and T56.

**Table 4:** Concentration (ng/ml) of plasma and urine caffeine of horses at T0 and T56

	Plasma		Urine	
Animal	T0	T56	T0	T56
**1**	1.5	55,143	14.5	94,202
**2**	4.3	56,844	18.1	57,038
**3**	4.2	57,890	8.6	79,946
**4**	3.7	44,986	11.9	59,178
**5**	4.5	46,275	15.5	62,103
**6**	30.2	48,248	13.1	54,002

**Mean**	8.1^a^*	51,564^b^	13.6^a^	67,744^b^
**St. Dev**.	10.9	5,708	3.2	15,859

*Different letters shown for each variable (plasma and urine) represent statistically significant differences (P < 0.001).

## Discussion

Coffee husk consumption by the horses was spontaneous and caused temporary intoxication, thus confirming the suspicions of the owners and veterinarians, who reported similar conditions in horses that ingested coffee husks used as bedding in their stalls. The signs observed were similar to those described in animals that were spontaneously intoxicated.

Coffee husk intake was responsible for the clinical intoxication observed in horses, and the improvement of signs was associated with discontinuation of the coffee husk supply. According to Barcelos et al. [22], the caffeine concentration in the husks of Arabica coffee varies between 0.5% and 1.3%, thus supporting the amount of caffeine found in the coffee husks used in this experiment. Caffeine was the substance responsible for the clinical signs observed, given that the mycotoxin analysis and the absence of insecticides in coffee husks excluded other conditions due to intoxication that exhibit neurological signs in horses, such as leukoencephalomalacia [23], and those that include signs of hyperexcitability, such as intoxication by chlorinated insecticides [24]. Furthermore, cattle have been shown to display signs of intoxication after one week of ingesting 3 kg, on average, of coffee husks used as bedding in their stalls [21]. Nevertheless, the addition of up to 1 kg of coffee husks with 0.97% caffeine in the diet of cattle did not produce clinical signs of intoxication [3,6].

The animals were not very interested in consuming coffee husks during the first hours after they were supplied; however, after ingesting the husks for the first time, the animals generally preferred them to hay. This behavior was also described by Nazário et al. [21]. It is worth noting that in the present experiment, the animals were fully adapted, and the supply of hay, in its own trough, was not discontinued; furthermore, hay consumption by the animals was within the levels recommended by the National Research Council (NRC) [25] for the species.

The ELISA method, which was used to quantify plasma caffeine levels, has been compared with the gas chromatography method in humans [26] and in horses [27], and it has been proven effective in quantifying the substance in both species. ELISA has the advantage of being cheap and practical; however, if the individual ingests supplements that contain other methylxanthines, cross-reactions may occur and the concentration of caffeine may be overestimated [26]. The ELISA kit used in this study had cross-reactivity of 24% with theobromine and 0.06% with theophylline. In the initial part of this experiment, animals were only given coast cross hay, which does not contain any substances belonging to the methylxanthine group, which can be demonstrated by the low concentration of caffeine detected in the plasma and urine at T0, thus excluding the likelihood of this interference. The high plasma caffeine levels detected at T56 was not influenced by cross-reactivity with theobromine and theophylline, since they were not detected in coffee husk by HPLC. It was previously described that caffeine is metabolized to many methylxanthine compounds, including theobromine and theophylline [11]. These substances may influence urine caffeine levels found in this present study at T56, however the high magnitude concentration increase (compared to T0) was mainly due to the caffeine present in the ingested coffee husk.

The bioavailability of caffeine in horses after oral administration was 39% [28], and as the total average caffeine consumption by the animals in the present study was 78 mg per kilogram of body weight (mg/kg BW), therefore, on average, approximately 30 mg/kg BW of caffeine were absorbed by the animals over 56 h. Behavioral changes occur in horses when plasma caffeine concentrations are greater than 2,000 ng/ml [11]. Similar results were also observed by Vickroy et al. [29], who reported an increase in motor activity in horses with plasma caffeine concentrations of 4,000 ng/ml. Therefore, the clinical signs observed in the horses participating in this study (compulsive walking, mydriasis, congested ocular mucosa and episcleral vessels and intense sweating) may reflect the high plasma concentrations of caffeine (51,564 ± 5,708 ng/ml) observed at T56. In humans, clinical signs of caffeine intoxication manifested after an intake of over 10 mg/kg BW [30], and a dose of 15 mg/kg BW of caffeine caused severe changes in the central nervous system (anxiety, delirium, vomiting and seizures) and circulatory system [31].

The clinical signs of intoxication ceased between 12 h (animals 3, 4 and 6) and 40 h (animals 1, 2 and 5) after access to coffee husks was discontinued, which is similar to the results found in cattle after spontaneous intake of coffee husks [21]. These authors reported that most animals showed rapid and complete remission of clinical signs between 3 and 4 h, whereas in other animals, signs only ceased 24 h after restricting access to coffee husks. Similar signs to the involuntary movements (dyskinesia) of the mouth and tongue shown by animals 1, 2, 3 and 5 were also described in humans after excessive caffeine intake and has been called "bucco-linguo-masticatory syndrome" [32]. The heart and respiratory rates and the rectal temperatures of animals increased after 36 h of supplying them with coffee husks, and unlike the neurological signs, these measurements only returned to normal values 64 h after discontinuing the coffee husk supply.

In our study, there were no residual sequelae or deaths; however, there are reports from professionals in the field that some intoxicated animals die when coffee husk intake is not discontinued. Nevertheless, it is emphasized that these reports do not constitute scientific proof; furthermore, the lethal dose of caffeine for horses was not found in the literature. In humans [33] and dogs [15], doses of 75 mg/kg BW and 140 mg/kg BW of caffeine, respectively, are considered lethal.

There was an increase in serum total protein, albumin and globulin at T56, which occurred as a result of the 5% dehydration that was clinically detected in four animals. This dehydration could be attributed to the excessive sweating observed at T56 and the lack of water consumption by horses exhibiting clinical signs. Hyperglobulinemia can also be observed during inflammation, but the serum iron concentration, an early indicator of inflammation in horses [34], was not affected and remained within normal parameters for horses.

Serum CK activity increased at T56 relative to T0, and in five animals, they were greater than those considered to be normal for the species (100 to 300 IU/l) [35]. The activity of this enzyme increases rapidly after muscle damage [36]. In the present study, this increase could have resulted from muscle tremors and increased motor activity, because the highest CK activities were observed in animals that exhibited this sign more intensely. The average serum concentration of AST, used to evaluate muscle and liver functions, was higher at T56 than at T0 (p = 0.03); however, it was still within the normal range for the species [35]. These values could have been higher and exceeded this threshold, given that the serum concentration of this enzyme, unlike CK, increases gradually and only reaches its peak 24 to 36 h after muscle damage [36]. Although AST concentrations also increase after liver damage, the average increase in AST concentration observed in the animals from this study suggested muscle damage, because the serum activity of GGT, another enzyme that assesses liver function, did not differ between T0 and T56 (p > 0.05). Furthermore, it is known that coffee intoxication can cause muscle damage; cases of rhabdomyolysis have been reported in humans as a result of caffeine overdose [37].

The serum urea concentration decreased at T56, but there was no significant difference between the two time periods. In contrast, serum creatinine significantly increased (p < 0.001) at T56 relative to T0. Creatinine is derived from muscle creatine, and intense muscle activity can explain the increased creatinine in this study [38].

The blood gas values at T56 revealed metabolic acidosis in animals 1, 2, 3 and 5, which had lower HCO_3 _values than the standard values for the species. However, caffeine stimulation of respiratory centers could have caused bronchodilation [28] and increased oxygen uptake [39], resulting in hyperpnea (an increase in respiratory rate and amplitude), which was observed between T48 and T60. This condition caused an increase in PO_2 _and SO_2_, as well as a reduction in pCO_2 _and tCO_2_, thus counteracting the metabolic acidosis with respiratory alkalosis. Although arterial blood gas is more representative for the variables of PO_2_, SO_2_, pCO_2 _and tCO_2 _[40], the blood gas changes described above were apparent, even in venous blood.

The levels of chloride and sodium electrolytes detected were not significantly different at T56 relative to T0; however, the levels of potassium and calcium were significantly reduced (p < 0.05). Similar results have been found by other authors who evaluated these ions after animals exercised and began sweating [41,42].

The relative polycythemia observed was associated with hemoconcentration (confirmed by the increase in serum TP at T56) and splenic contraction, which releases a large number of erythrocytes into the circulation [36]. In a study performed by Kurosawa et al. horses that were given caffeine and subjected to exercise had a greater packed cell volume than horses that were not given caffeine [39]. Caffeine increases the release of catecholamines, especially epinephrine, which stimulates splenic contraction, thus increasing Htc [36,43]. Furthermore, caffeine can induce diuresis in horses [44], although urine production was not measured in the present study, this effect might have contributed to the Htc increase.

The detection of caffeine in sport horses has been considered doping by the International Equestrian Federation; however, in addition to the intentional administration of caffeine with the intent of improving performance, caffeine can also be ingested as a result of feed contamination. Currently, caffeine is not on the list of prohibited substances, but it is on a monitoring list, and investigations are only conducted when high concentrations are detected [45]. The high concentration of caffeine found in the plasma and urine in this study could be characterized as doping, even if intake was accidental. Compared to T0, the caffeine levels found at the onset of clinical signs (T56) were, on average, 6,366 times greater in the plasma and 4,981 times greater in the urine.

## Conclusion

It is concluded that coffee husks are toxic for horses and must not be used as bedding or be provided in horses' feed; furthermore, although a lethal dose for horses is not described in the literature, it is probably that excessive and continuous consumption of coffee husks could lead to the death of animals as a result of severe cardio-circulatory changes and electrolyte imbalances.

## Competing interests

The authors declare that they have no competing interests.

## Authors' contributions

ASB and CAH designed the study. FLC described natural poisoning cases. DJZD, JPOF and TK performed the clinical examination and collected samples. ASB, DJZD, JPOF and JPAJ performed laboratorial analysis. ASB, DJZD, FRC, CAH and JPOF reviewed the literature and prepared the manuscript. All authors read and approved the final manuscript.

## Authors' information

DJZD, JPOF, TK, CAH and ASB are from College of Veterinary Medicine and Animal Science, Univ. Estadual Paulista (Unesp), Botucatu, Brazil. FLC is from Faculdade de Medicina Veterinária, Fundação Pinhalense de Ensino, Brazil. FRC is from Universidade Federal de Campina Grande, Patos, Brasil. JPAJ is from Department of Immunology and Microbiology, Biosciences Institute of Botucatu, Univ. Estadual Paulista (UNESP), Botucatu, SP., Brazil.

## Supplementary Material

Additional file 1 **Horse number 1, 56 h after initial coffee husk intake (T56), showing anxious expression, excitability, restlessness, involuntary tremors and constant movement of the lips, flaring nostrils, involuntary muscle tremors, and sweating**. Horse number 5 at T56, showing excitability and restlessness at the end of this video. Click here for file
